# Ultra-widefield fundus autofluorescence in age-related macular degeneration

**DOI:** 10.1371/journal.pone.0177207

**Published:** 2017-06-01

**Authors:** Abhilash Guduru, David Fleischman, Sunyoung Shin, Donglin Zeng, James B. Baldwin, Odette M. Houghton, Emil A. Say

**Affiliations:** 1 Univeristy of North Carolina School of Medicine, Chapel Hill, North Carolina, United States of America; 2 Department of Ophthalmology, Kittner Eye Center, University of North Carolina, Chapel Hill, North Carolina, United States of America; 3 Department of Statistics, University of Wisconsin, Madison, Wisconsin, United States of America; 4 Department of Biostatistics and Medical Informatics, University of Wisconsin, Madison, Wisconsin, United States of America; 5 Department of Ophthalmology, Storm Eye Institute, Medical University of South Carolina, Charleston, South Carolina, United States of America; Universita degli Studi di Firenze, ITALY

## Abstract

**Background:**

Establish accuracy and reproducibility of subjective grading in ultra-widefield fundus autofluorescence (FAF) imaging in patients with age-related macular degeneration (AMD), and determine if an association exists between peripheral FAF abnormalities and AMD.

**Methods:**

This was a prospective, single-blinded case-control study. Patients were consecutively recruited for the study. Patients were excluded if there was a history of prior or active ocular pathology other than AMD or image quality was insufficient for analysis as determined by two independent graders. Control patients were those without any evidence of AMD or other ophthalmic disease apart from cataract. Using the Optos 200Tx (Optos, Marlborough, MA, USA), a ResMax central macula and an ultra-widefield peripheral retina image was taken for each eye in both normal color and short wavelength FAF. Ultra-widefield photographs were modified to mask the macula. Each ResMax and ultra-widefield image was independently graded by two blinded investigators.

**Results:**

There were 28 AMD patients and 11 controls. There was a significant difference in the average age between AMD patients and control groups (80 versus 64, respectively *P*<0.001). There was moderate, statistically significant agreement between observers regarding image interpretation (78.4%, K = 0.524, *P<*0.001), and 69.0% (K = 0.49, *P<*0.001) agreement between graders for FAF abnormality patterns. Patients with AMD were at greater risk for peripheral FAF abnormalities (OR: 3.43, *P* = 0.019) and patients with FAF abnormalities on central macular ResMax images were at greater risk of peripheral FAF findings (OR: 5.19, *P* = 0.017).

**Conclusion:**

Subjective interpretation of FAF images has moderate reproducibility and validity in assessment of peripheral FAF abnormalities. Peripheral FAF abnormalities are seen in both AMD and control patients. Those with AMD, poor visual acuity, and macular FAF abnormalities are at greater risk.

## Introduction

Age-related macular degeneration (AMD) is a leading cause of adult blindness.[1] In fact, a global meta- analysis of available population-based studies published between 1990 and 2010 reported that 2.1 million people were blind and 6.0 million people were visually impaired in 2010 from AMD.[1] Despite its high prevalence, the exact pathogenesis and causes of AMD are yet to be fully understood. Until recently, progression and severity of the disease is characterized by the changes that happen in the macula as established the by the International Age-related Macular Degeneration Epidemiological Study Group.[2]

FAF is an imaging modality that allows subjective assessment of the overall health of the retinal pigment epithelium as reflected by the amount of lipofuscin component A2E.[3,4] The presence of FAF abnormalities in the macula has been extensively studied and well documented in eyes with AMD.[3–6] More recently, several studies have used ultrawidefield imaging to demonstrate increased peripheral FAF abnormalities in AMD, as well as other conditions such as central serous retinopathy, and retinal vascular disease.[7–9] Reznicek et. al. quantified the short- wavelength autofluorescence intensity in eyes with AMD and found that this was significantly increased indicating a more widespread RPE disease than previously thought.[8] AREDS2 had an ancillary study using ultra-widefield imaging and found 81% had an irregular fundus autofluorescence (FAF) pattern, while 67% specifically had reticular pigmentation.[10–12] More importantly, these peripheral changes have been shown in a separate report using ultra-widefield imaging to correlate with specific single nucleotide polymorphisms (SNPs) that predispose to neovascular AMD such as CFH, ARMS2, and HTRA1.[13] However, analysis of peripheral abnormalities in ultra- widefield imaging is inherently biased in patients with AMD without masking the macula FAF abnormalities.

The goal of this study was to establish accuracy and reproducibility of subjective grading in ultra-widefield FAF imaging in patients with AMD. Secondarily, the association between peripheral FAF abnormalities and AMD is also studied in comparison to normal controls. A systematic, blinded, prospective case-control analysis with two independent graders was performed to limit bias and assess for true peripheral FAF changes in these patients.

## Methods

### Study and subjects

This study was approved by the Institutional Review Board (University of North Carolina at Chapel Hill) and met the Tenets of the Declaration of Helsinki. All patients provided an informed written consent to participate in this investigation. This was a single blinded prospective case-control study comparing ultra-widefield autofluorescence findings in patients with AMD and normal controls. Patients were recruited prospectively in a consecutive manner between January 2012 and February 2012. In the AMD group, patients were excluded if previous retinal pathology was present other than AMD. Patients in the control group were excluded if they have any ocular pathology other than mild nuclear sclerosis and cataracts.

### Image acquisition and analysis

**Fig 1:**
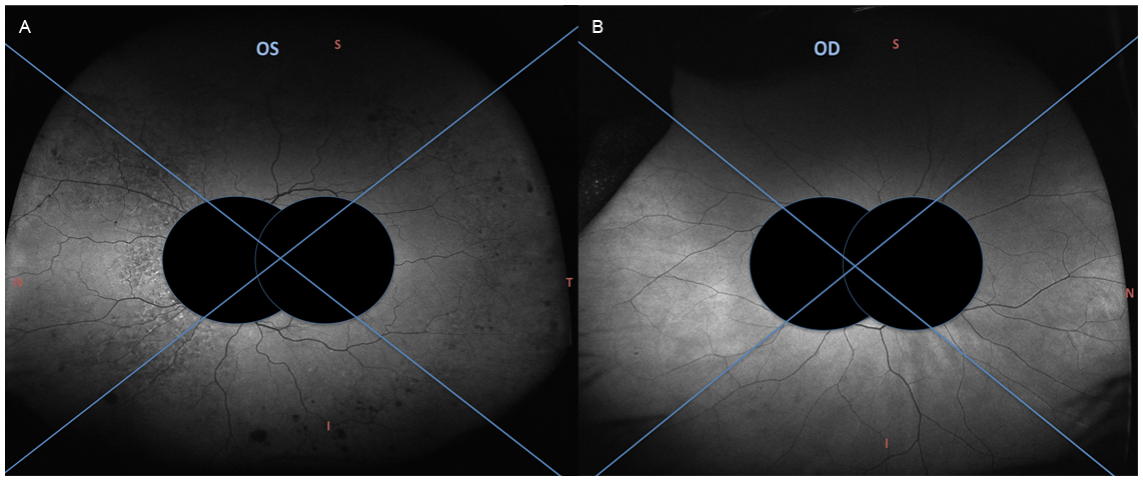
Examples of masked widefield image for grading. A: Consensus agreement between both graders that the image was characterized as abnormal, with both patterns of autofluorescence (hyper- and hypo-autofluoresnce), and located in all four quadrants. B: Consensus agreement between both graders that this was a normal autofluorescence image.

**Fig 2:**
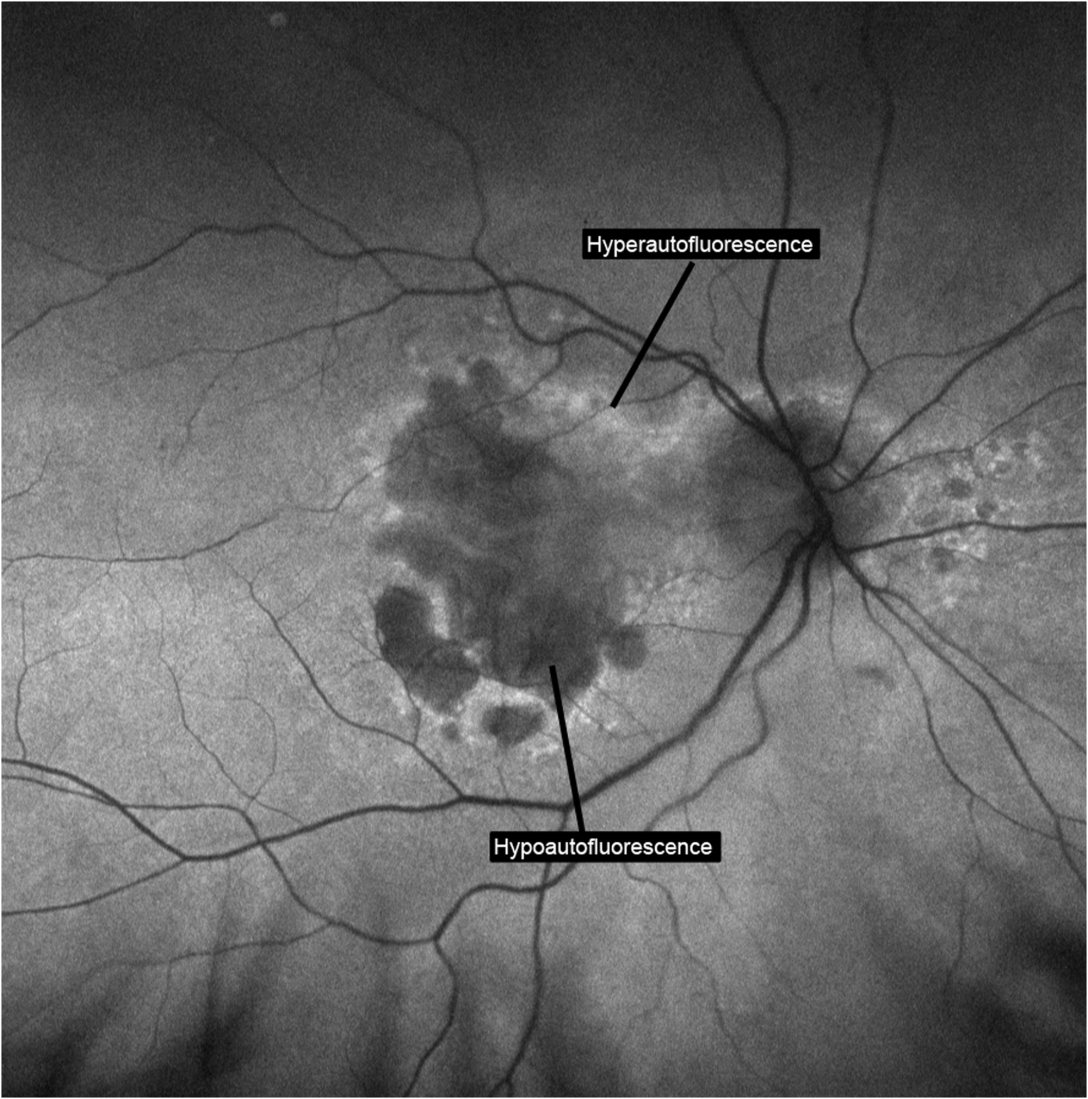
An example of a central ResMax with pattern abnormalities (hypo- and hyper-autofluorescence, both labeled).

Each patient had a ResMax central macula and ultra-widefield peripheral retina image using the Optos200Tx (Optos, Marlborough, MA, USA) with both standard color and short-wavelength autofluorescence imaging, a means to visualize the peripheral retina.[14] Each eye was treated as an independent observation, and both eyes nested within each patient were included for analysis. Two blinded expert graders (EAS, OMH) independently evaluated each image with masked macula. (Fig 1A and 1B) Each grader was asked to determine image quality (gradable or ungradable), autofluorescence interpretation (normal or abnormal), and type of autofluorescence abnormality if present (hypoautofluorescence or hypoAF, hyperautofluorescence or hyperAF, or both). Further, each single image was separated into four quadrants (superior, inferior, nasal, temporal) and each grader was asked to determine autofluorescence pattern for each quadrant (hypoAF, hyperAF, both, or none) (Figs 1A, 1B and 2). Both graders had to agree that the image quality was gradable in order for the image to be included in statistical analysis. The macula was also graded independently of the periphery and similarly assessed for autofluorescence abnormalities.

### Statistical analysis

Statistical software R version 3.2.1 was used for statistical analysis. The dataset used in this study is within the supporting information (S1 Dataset). The association of peripheral FAF abnormalities with AMD status was tested in a model-based analysis: a logistic regression model with generalized estimating equation (GEE). GEE is a useful estimation method to model repeatedly measured responses with valid statistical inference under a potentially incorrectly specified correlation structure between the repeated responses.[15] In the model, the responses were the autofluorescence abnormalities assessed by the two graders, and the covariates were grader, eye graded (OS or OD), and AMD status. To determine graders’ agreement in the assessment of peripheral FAF abnormalities, we constructed frequency tables of each grader’s assessment and calculated Cohen’s kappa. Graders’ agreement was also evaluated per quadrants. We next fitted another logistic regression with GEE to discover associations between peripheral FAF abnormalities and clinical factors. The clinical features include patients’ age, sex, AMD family history, smoking/alcohol history, and visual acuity. To assess the correlation between peripheral and macular FAF abnormalities, we constructed a descriptive 2 by 2 contingency table for the abnormalities of eyes whose FAF interpretation was agreed by both graders, and performed Fisher’s exact test. Further, logistic regression analysis with GEE was performed to confirm any association between both macular and peripheral FAF abnormalities.

## Results

There were 28 AMD patients and 11 control patients included in the study. Patient demographics are presented in Table 1. Mean patient age of the AMD group was 80 years, which is significantly older than 64 years in the control group (*P*<0.001). Among the 56 eyes of 28 patients in AMD group, 33 eyes had neovascular AMD, while 23 had non-neovascular AMD. Further, AMD group has larger number of smokers and positive AMD family history; however, their statistical significance is not achieved under the level of 0.05 from two-sample test of proportions (*P* = 0.059, *P* = 0.074). We initially gathered a total of 78 eyes and 156 images. After excluding 33 poor and ungradable images and 11 images from patients with visual acuity of counting fingers, 112 images from 61 eyes were included for analysis (51 eyes with 2 gradings and 10 eyes with 1 grading). The images are the data points of our statistical analyses.

**Table 1:** Ultra-widefield short wavelength fundus autofluorescence in patients with age-related macular degeneration. Patient demographics.

		Control Group, n = 11	AMD Group, n = 28	p-value
**Mean age**		64	80	<0.001
**Sex**				0.713
	Male	3	11	
	Female	8	17	
**Smoking History**				0.059
	Yes	1	13	
	No	9	15	
	NA*	1	0	
**Family History of AMD**				0.074
	Yes	0	9	
	No	9	17	
	NA*	2	2	
**Type of AMD, n = 56 eyes**				
	Neovascular	0	33	not compared
	Non-neovascular	0	23	

*NA indicates that the information is not available.

We fitted all 112 images to a logistic regression with GEE in order to assess association between peripheral FAF status and AMD. The logistic regression analysis with GEE showed that the AMD group had greater risk (OR: 3.43, CI: 1.23–9.56, *P* = 0.019) for peripheral FAF abnormalities compared to normal control.

Inter-observer agreement regarding interpretation of ultra-widefield FAF images is presented in Tables 2 and 3. In general, both graders agreed in determination of normal versus abnormal images (K = 0.524), and there was 69.0% (K = 0.49) agreement between graders regarding FAF abnormality patterns. The Kappa statistics between 0.4 and 0.6 indicate both graders’ determinations have a moderate agreement.[16] Most common peripheral FAF abnormal patterns are hyperAF and both hyper- and hypoAF abnormalities from the agreed pattern grading (60% and 30% respectively). We further evaluated a general inter-observer variability per quadrants with the 29 eyes that were deemed abnormal by both graders (Table 4). While the agreement was fair for the nasal (K = 0.378), and the temporal quadrants (K = .253), the agreement was moderate for the inferior (K = 0.529), and superior (K = 0.59) quadrants. Based on the agreed FAF grading, the nasal quadrant was the most affected by peripheral FAF abnormalities (91.7%), followed by the temporal quadrant (73.7%), and the inferior and superior quadrants were least affected (45.5% and 43.5% respectively).

**Table 2:** Ultra-widefield short wavelength fundus autofluorescence in patients with age-related macular degeneration.

Agreement in presence of any peripheral fundus autofluorescence abnormalities								
		Grader 2			Observed Agreement	Expected Agreement	Kappa*	p-value
		Normal	Abnormal	Total				
Grader 1	Normal	11	10	21	78.4%	54.7%	0.524	<0.001
	Abnormal	1	29	30				
	Total	12	39	51				

*Cohen’s kappa, based on the frequency table of the graders’ assessments

**Table 3:** Ultra-widefield short wavelength fundus autofluorescence in patients with age-related macular degeneration. Inter-observer variability of peripheral autofluorescence abnormalities.

Agreement in patterns of peripheral fundus autofluorescence abnormalities									
		Grader 2				Observed Agreement	Expected Agreement	Kappa*	p-value
		HyperAF	HypoAF	Both	Total				
Grader 1	HyperAF	12	0	5	17	69.0%	39.1%	0.49	<0.001
	HypoAF	0	2	3	5				
	Both	1	0	6	7				
	Total	13	2	14	29				

**Table 4:** Ultra-widefield short wavelength fundus autofluorescence in patients with age-related macular degeneration. Inter-observer agreement of peripheral autofluorescence abnormalities per quadrant.

**Agreement in presence of inferior peripheral fundus autofluorescence abnormalities**								
		Grader 2			Observed Agreement	Expected Agreement	Kappa*	p-value
		Normal	Abnormal	Total				
Grader 1	Normal	12	6	18	75.9%	48.8%	0.529	0.002
	Abnormal	1	10	11				
	Total	13	16	29				
**Agreement in presence of nasal peripheral fundus autofluorescence abnormalities**								
		Grader 2			Observed Agreement	Expected Agreement	Kappa*	p-value
		Normal	Abnormal	Total				
Grader 1	Normal	2	5	7	82.8%	72.3%	0.378	0.009
	Abnormal	0	22	22				
	Total	2	27	29				
**Agreement in presence of temporal peripheral fundus autofluorescence abnormalities**								
		Grader 2			Observed Agreement	Expected Agreement	Kappa*	p-value
		Normal	Abnormal	Total				
Grader 1	Normal	5	7	12	65.6%	53.9%	0.253	0.154
	Abnormal	3	14	17				
	Total	8	21	29				
**Agreement in presence of superior peripheral fundus autofluorescence abnormalities**								
		Grader 2			Observed Agreement	Expected Agreement	Kappa*	p-value
		Normal	Abnormal	Total				
Grader 1	Normal	13	5	18	79.3%	49.6%	0.59	<0.001
	Abnormal	1	10	11				
	Total	14	15	29				

*Cohen’s kappa, based on the frequency table of the graders’ assessments

Subgroup analysis was performed to determine factors associated with peripheral FAF abnormalities. After excluding 17 images with missing clinical history, we considered 95 images and used GEE in a logistic regression with the clinical features mentioned above. From the model, peripheral FAF abnormalities were associated with age (OR = 1.15 per year increase in age, CI: 1.07–1.25, *P<*0.001) and poor visual acuity (OR: 0.737 per 0.1 logMAR increase, CI: 0.592–0.918, *P* = 0.007). History of smoking was not considered to be associated with peripheral FAF abnormalities at the significance level of 0.05.

Next, we analyzed the relationship in FAF abnormalities between the central macula and peripheral retina with 104 gradable images for both FAF assessments out of the 112 images. Among them, 88 images were from 44 eyes graded by both readers and 16 images were from 16 eyes graded by only one reader. We considered 32 eyes having agreed FAF interpretation out of the 44 eyes for Fisher’s exact test. The analysis showed significant statistical association between macular and peripheral FAF abnormalities (*P* = 0.01). This association was further corroborated by GEE in a logistic regression model with the 104 images, wherein eyes with macular FAF abnormalities are at a greater risk for peripheral FAF abnormalities (OR: 5.19, CI: 1.34–20.1, *P* = 0.017).

We performed secondary analysis in order to see how the association between AMD status and peripheral FAF abnormalities is changed when age is taken into account. We refitted the 112 images to age-adjusted logistic regression model with GEE. From the fitting, the OR of peripheral FAF abnormalities given AMD versus controls is 1.46 (CI: 0.36–5.96). The change in the ORs is 57.5% compared to the OR of 3.43 from the primary fitting. The noticeable change from the analysis also shows the need for age-matched controls in future studies.[17]

## Discussion

This study confirms previous reports indicating the higher incidence of peripheral FAF abnormalities in patients with AMD. The present and the previous reports indicate that patients’ age is associated with both AMD status and peripheral FAF abnormalities, although this might also confound their association considering that we did not age-match our controls.[7] The peripheral FAF abnormalities were appreciated with moderate agreement between two masked independent observers, even when assessing FAF abnormalities per quadrant. Compared to the previous report by Tan et. al., our graders were masked both to clinical features, as well as macular FAF abnormalities.[18] Graders from that report had high agreement regarding assessment of FAF abnormalities with K = 0.95 for the type of FAF abnormality and K = 0.91 for the extent of these abnormalities, similar to the agreement between observers in the present report despite masking the macular FAF features. Our study has shown that subjective grading has moderate validity and reproducibility in FAF assessment. Similarly, results from a prior study on subjective determination of FAF abnormalities in ultra-widefield FAF imaging should be considered data that corroborate increased peripheral FAF abnormalities in AMD. However, it is important to keep in mind that both studies have only two graders, which may lead to higher chance of variation in the assessments.[19] Further, FAF abnormalities in general are not binary (black and white) and rather, appear in various grayscale intensities. Hence, in order to enhance the reliability of the subjective grading, future studies are suggested to have more graders and provide graders’ adequate training on image interpretation.

FAF is due to the inherent fluorophore, lipofuscin A2E, found in the retinal pigment epithelium (RPE). Abnormalities on FAF can be seen in AMD, intraocular tumors, and retinal dystrophy as a result of oxidative damage, retinal degeneration, or inflammatory disruption of the RPE.[20,21] Increased peripheral FAF abnormalities in this study is a reflection of a more widespread RPE insult in AMD, rather than localized macular involvement.

From a molecular standpoint, proteonomic studies in AMD also show that pathologic proteins like VEGF involve the peripheral retina in 40%, indicating that AMD is a more widespread disease than previously thought.[22–24]

Previous reports on peripheral FAF changes in patients with AMD show a greater number of hyperAF compared to hypoAF abnormalities with limited significance.[18,22] The differences in pattern likely depend on disease severity, duration, and individual patient phenotype. Comparing peripheral FAF to central FAF changes, eyes that have early and mild AMD present with predominately hyperAF changes, with conversion to hypoAF as disease progression occurs, ultimately culminating in geographic atrophy represented as complete hypoAF.[25] Witmer et. al. revealed the predominant pattern of peripheral FAF abnormality in AMD is the granular pattern and this was corroborated by a separate study by Tan et. al.[18, 22] Other patterns of peripheral FAF abnormalities include mottled or nummular patterns, and in 30%, multiple patterns can be seen in the same eye.[18] These studies further categorized eyes based on severity of disease and other clinical features, such as drusen and presence of neovascularization.[18,22] They showed peripheral FAF changes are seen most frequently in eyes with neovascular AMD (86%), followed by non- neovascular AMD (73%), and least in normal eyes (18%).[18] Further, they found the nasal quadrant to be most frequently involved overall among all eyes (55%), followed by the temporal (38%), superior (33%), and inferior (24%), with a 90% concordance between eyes.[18] These are similar with our results reported in the previous section to an extent, with the nasal quadrant being most affected, followed by the temporal, inferior, and superior. It is currently unknown why the nasal quadrant is more often affected and future studies may address this finding. The small sample size in the present series did not allow for evaluation of specific clinical features associated with peripheral FAF abnormalities, but the greater number of hyperAF and both hyper- and hypoAF abnormalities in the peripheral retina seen in this study are in agreement with previous publications. It is also important to note that peripheral FAF abnormalities can be seen with other conditions, and even normal eyes. In fact, 18–20% of normal eyes can have peripheral FAF abnormalities in one study, but this is generally less.[7,22]

Lastly, it is important to understand the influence of demographic features and clinical findings with peripheral FAF abnormalities. Our study showed that poor vision and increasing age significantly correlated with a higher incidence of abnormalities, whereas family history, smoking, and alcohol consumption significantly did not affect peripheral findings. Others studies have also found that being older (OR: 4.9–11.3, depending on age quartile) and female (OR: 2.36) had a higher degree of FAF, and that coexisting conditions such as hypertension, diabetes mellitus, or ischemic heart disease did not affect peripheral findings significantly.[7,22] The association of logMAR visual acuity to these changes in this study is intriguing, and is likely related to the generally worse visual acuity in eyes with neovascular AMD. It is interesting to note that smoking had a trend of association with peripheral FAF, albeit not statistically significant. A stronger relationship would not have been surprising due to the vascular insult that tobacco inhalation would likely have on the RPE, while increasing age is a strong factor for development of AMD, which is independently associated with peripheral FAF abnormalities. Further studies need to be conducted on larger populations to further characterize this relationship, as this is outside the scope of the present study.

In summary, subjective grading of peripheral FAF abnormalities on ultra-widefield FAF imaging is has moderate validity and reproducibility. The present report validates previous studies and supports data indicating widespread effects on the RPE in patients with AMD. Future avenues on ultra-widefield FAF imaging include the influence of smoking, age, and other clinical features on the pattern and location of peripheral retinal FAF abnormalities.

## Supporting information

S1 Dataset Study data points. (CSV)
